# Diagnostic utility of capnography in emergency department triage for screening acidemia: a pilot study

**DOI:** 10.1186/s12245-024-00631-3

**Published:** 2024-04-22

**Authors:** Paul Peng, Alex F. Manini

**Affiliations:** 1 Department of Emergency Medicine, The State University of New Jersey, 08901 Rutgers, New Brunswick, NJ United States of America; 2 Division of Medical Toxicology, Department of Emergency Medicine, Icahn School of Medicine at Mount Sinai, 10029 New York, NY United States of America

**Keywords:** Capnography, End-tidal CO_2_, Respiration, Acidemia, Vital signs

## Abstract

**Background:**

Capnography is a quantitative and reliable method of determining the ventilatory status of patients. We describe the test characteristics of capnography obtained during Emergency Department triage for screening acidemia.

**Results:**

We performed an observational, pilot study of adult patients presenting to Emergency Department (ED) triage. The primary outcome was acidemia, as determined by the basic metabolic panel and/or blood gas during the ED visit. Secondary outcomes include comparison of estimated and measured respiratory rates (*RR*), relationships between end-tidal CO_2_ (*EtCO*_*2*_) and venous partial pressure of *CO*_*2*_, admission disposition, in-hospital mortality during admission, and capnogram waveform analysis. A total of 100 adult ED encounters were included in the study and acidemia ($$ \left[HC{O}_{3}^{-}\right]\le 22 \text{mEq/L}$$ or $$ pH< 7.35$$) was identified in 28 patients. The measured respiratory rate (20.3 ± 6.4 breaths/min) was significantly different from the estimated rate (18.4 ± 1.6 breaths/min), and its area under the receiver operating curve (c-statistic) to predict acidemia was only 0.60 (95% CI 0.51–0.75, *p* = 0.03). A *low* end-tidal CO_2_ (*EtCO*_*2*_ *< 32* mmHg) had positive (LR+) and negative (LR−) likelihood ratios of 4.68 (95% CI 2.59–8.45) and 0.34 (95% CI 0.19–0.61) for acidemia, respectively—corresponding to sensitivity 71.4% (95% CI 51.3–86.8) and specificity 84.7% (95% CI 74.3–92.1). The c-statistic for *EtCO*_*2*_ was 0.849 (95% CI 0.76–0.94, *p* = 0.00). Waveform analysis further revealed characteristically abnormal capnograms that were associated with underlying pathophysiology.

**Conclusions:**

Capnography is a quantitative method of screening acidemia in patients and can be implemented feasibly in Emergency Department triage as an adjunct to vital signs. While it was shown to have only modest ability to predict acidemia, triage capnography has wide generalizability to screen other life-threatening disease processes such as sepsis or can serve as an early indicator of clinical deterioration.

**Supplementary Information:**

The online version contains supplementary material available at 10.1186/s12245-024-00631-3.

## Background

Respiratory rate (*RR*), the most oft-neglected vital sign, is a window into the body’s ventilatory and metabolic status [1]. An even more accurate reflection of ventilation is obtained with capnography, which is a noninvasive measurement of carbon dioxide as a function of time, $$ {P}_{E}{CO}_{2}$$

$$ {P}_{E}{CO}_{2} \propto \frac{\dot{V}{CO}_{2}}{RR\times {V}_{T}}$$

where $$ \dot{V}{CO}_{2}$$ is the metabolic production of $$ {CO}_{2}$$ and $$ {V}_{T}$$ is the tidal volume [2]. A capnogram can instantaneously identify apnea (*RR* ∼ 0), identify impending respiratory depression ($$ {P}_{E}{CO}_{2}>$$ 50 mmHg), or trigger further investigation of increased respiratory drive ($$ \uparrow $$*RR*). Capnography is currently standard practice in the Emergency Department (ED) during procedural sedation, confirming a definitive airway, and for assessing the metabolic effectiveness of cardiopulmonary resuscitation [3–7]. Recent studies have shown that capnography to be a useful adjunct in evaluating other conditions have such as sepsis, trauma, pulmonary embolism, and diabetic ketoacidosis [8–15]. In particular, the end-tidal $$ {CO}_{2}$$ (EtCO_2_) which is the maximum number of $$ {P}_{E}{CO}_{2}$$ at end expiration, correlates linearly with the body’s serum bicarbonate. As acidemia occurs via lactic or ketoacidosis, serum bicarbonate *decreases* and there is greater respiratory drive ($$ \uparrow $$*RR*, rapid, shallow breathing) to compensate—resulting in a *lower* EtCO_2_ [16, 17]. While most studies have focused on changes in EtCO_2_ as it relates to pathophysiology, it is currently not known if an accurate determination of a quantitative capnogram has any clinical utility for screening disease in ED triage.

This study’s goal is to examine the clinical impact of a “capnograph profile” in triage for screening acidemia, a common acid-base abnormality that could signify lactic acidosis from sepsis, ketoacidosis, or renal failure. We explore the feasibility of recording a 10-second capnogram (which includes *EtCO*_*2*_, *RR*, and a $$ {P}_{E}{CO}_{2}$$ waveform) at the same time that vital signs are obtained. An accurate respiratory rate alone instead of an estimated number is hypothesized to add validity to triage vital signs. Using serum acid-base status as the gold standard, we further describe the test characteristics of screening acidemia. We also describe the utility of qualitative capnogram waveform analysis.

## Methods

This was a prospective, observational pilot study at an urban tertiary care center with more than 110,000 ED visits annually. Subjects were awake, cooperative, adult ED patients who were Emergency Severity Index (ESI) level 3 and able to give verbal consent. The study setting was the triage room of the ED during vital sign measurement. A convenience sample of participants were approached between January 2019 and February 2020 during peak hours of ED operation, approximately 12:00PM—4:00PM. The study protocol was approved by the Institutional Program for the Protection of Human Subjects and was also performed in accordance with the ethical standards as laid down in the 1964 Declaration of Helsinki and its later amendments or comparable ethical standards.

**Fig. 1:**
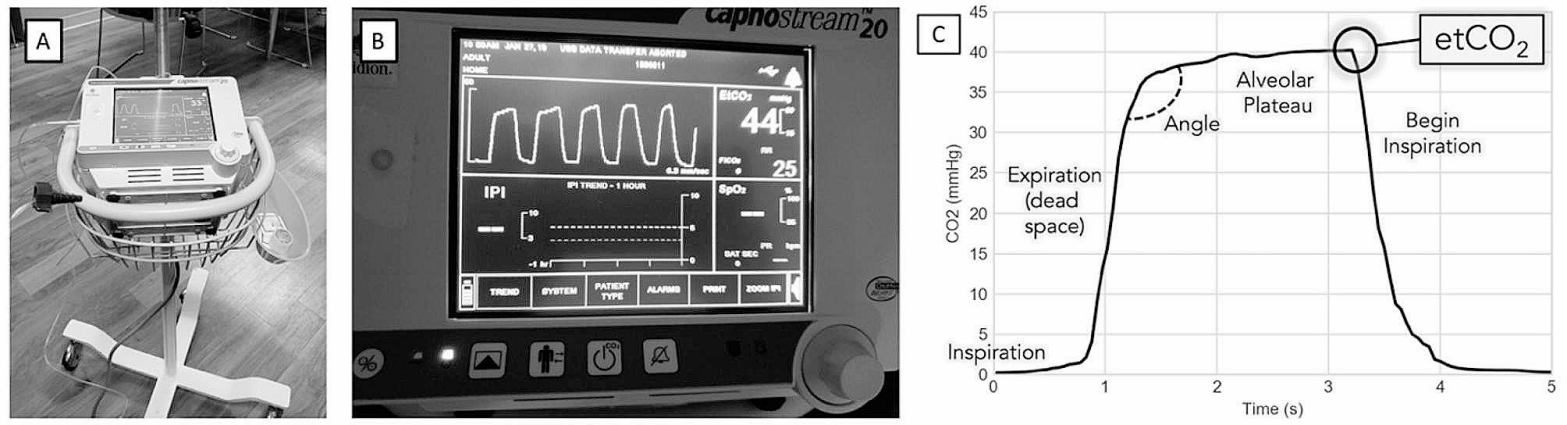
A *Capnostream 20™* portable respiratory monitor was **(A)** mounted on a small-footprint, movable pole. **(B)** Real-time display of *P*_*E*_*CO*_*2*_ as a function of time (capnogram) with values (at right) of *EtCO*_*2*_ = 44 mmHg and *RR* = 25 breaths/min. **(C)** Capnogram indicating the phases of inspiration and expiration. The *greater the angle*, the greater the degree of V/Q mismatch

Our intervention was a nasal cannula attached to a patient and connected to a portable capnometer (*Capnostream 20™*) mounted on a mobile apparatus (Fig. 1). It was chosen on the basis of low-cost and availability; the authors declare no conflicts of interest and the manufacturer had no involvement with the conception, design, or analysis of this study. This capnometer operated as a side stream device, which samples the respiratory circuit via a small tube and is easily applied via a nasal cannula. In contrast, mainstream devices have analyzers directly in the path of the breaths, can perform volumetric analysis, but are more expensive and bulkier. A new nasal cannula was used for each subject. Before implementation, the capnometer was registered at the hospital, calibrated against standard atmospheric gases, and verified to produce identical measurement values as compared to rack-mounted anesthetic gas modules. Acquisition of a capnogram added approximately 2 min to the measurement of vital signs, based on the investigators’ anecdotal experience.

Individuals were excluded from the study if they declined the intervention, left after triage, were upgraded directly to critical care resuscitation upon discovery of a severely abnormal vital sign such as hypotension (systolic blood pressure < 90 mmHg) or tachycardia, or if no basic metabolic panel or venous blood gas was drawn during the ED encounter. For patients who are not in shock, venous pH, bicarbonate, and base excess have sufficient agreement with arterial blood gases [18]. In order to prevent selection bias, participants were approached without prior knowledge of the chief complaint. Recruitment continued until the target *n* = 100 was reached, which was estimated by power analysis for detecting acidemia. Inclusion into the study required a serum bicarbonate $\left[HC{O}_{3}^{-}\right]$ level or *pH* value as the gold standard for diagnosing acidemia.

The *Capnostream 20™* capnometer in this study utilizes non-dispersive infrared spectroscopy to measure the amount of *CO*_*2*_ during each exhaled breath (*P*_*E*_*CO*_*2*_) as well as measuring the amount of *CO*_*2*_ at end-exhalation (*EtCO*_*2*_). The *P*_*E*_*CO*_*2*_ waveform is sampled with a resolution of 0.1 mmHg, and average respiratory rate has an accuracy of ±1 breath/min. An additional peripheral oxygen saturation measurement capability on the capnometer was not used in this study. After a stable *P*_*E*_*CO*_*2*_ waveform was displayed on the capnometer, a ten-second capnogram that also included average *RR* and *EtCO*_*2*_ was recorded. The capnometer’s measured *RR* value was not documented in the electronic health record, and the measurement protocol did not influence the acquisition of the other vital signs obtained in triage. Furthermore, only triage personnel were aware of the study protocol, and respiratory profile values (*P*_*E*_*CO*_*2*_ waveform, *RR*, and *EtCO*_*2*_) were not available to the ED providers who subsequently cared for the patient. Laboratory values were obtained by chart review of routine clinical care as indicated by a patient’s clinical course, and were independent on the time of capnography measurement.

The primary outcome of this study is the presence of acidemia during the ED encounter. In this study, acidemia was defined by either $$ \left[HC{O}_{3}^{-}\right]\le 22 mEq/L$$ or $$ pH< 7.35$$ [19]. The normal range of total bicarbonate was defined as $$ \left[HC{O}_{3}^{-}\right]=23-29 mEq/L$$, based on the study institution’s Chemistry Clinical Services reference standards and consistent with common reference literature. Secondary outcomes include alkalemia (defined as $$ \left[HC{O}_{3}^{-}\right]\ge 29 mEq/L$$ or $$ pH> 7.45$$) [19], admission disposition, in-hospital mortality after admission, septic shock, and qualitative analysis of abnormal capnogram waveforms. After completion of study enrollment, we abstracted further clinical data—including demographics, comorbidities—from the electronic health record. Statistical analyses were performed on the respiratory profile data to determine the degree of agreement between estimated and measured respiratory rates (*RR*) as well as the error in regression analysis with theoretical physiologic relationships among end-tidal CO_2_ (*EtCO*_*2*_), *RR*, and venous partial pressure of *CO*_*2*_ (*P*_*v*_*CO*_*2*_).

Patient and members of the public were not involved in the design, management, or conduct of this observational study. We carefully assessed the effect of this intervention on the patients as well as the nursing staff to minimize the burden on all those who were involved. We intend to share the results of the study to emergency department providers, and seek patient involvement in future directions of this research.

Patient characteristics are summarized with descriptive statistics (including means and standard deviations versus medians and interquartile ranges for parametric and non-parametric data, respectively). Statistical agreement between estimated and measured *RR* was evaluated using a Bland-Altman plot. Physiologic relationships were modeled using linear-regression with the Python scikit-learn library [20]. The receiver operating curve (ROC) for predicting acidemia was calculated using the IBM SPSS Statistics software package, and optimal cutoffs for *RR* and *EtCO*_*2*_ were calculated with the Youden index. Test characteristics are reported as sensitivity, specificity, positive and negative predictive values (assuming a 25% prevalence of acidemia), and positive and negative likelihood ratios, with respective 95% confidence intervals (CI). Accuracy of *RR* and *EtCO*_*2*_ for predicting acidemia was calculated with the equation, $Sens\times prev+Spec\times (1-prev)$. Sample size was calculated *a priori* using the *R* statistical computing software and *pwr* library package.^23^ A total sample size of 100 (with at least 20 in the acidemia arm) was calculated such that a test of means with a significance level of 0.05 has an 80% power to detect acidemia with effect size 0.7 (corresponding to a 2 mEq/L difference in $\left[HC{O}_{3}^{-}\right]$.

## Results

As shown in the flow diagram of the study in Fig. 1, a total of 131 patients were approached for this study and 100 met eligibility criteria for inclusion. Baseline characteristics of the patients are shown in Table 1. Subjects had a moderate burden of comorbidities and 40% required hospital admission. Acidemia was diagnosed in 28 patients, and 2 developed septic shock while another 2 patients expired during the associated hospitalization.

**Table 1:** Baseline Characteristics

Characteristics	Included (*n* = 100)
Epidemiologic Data	Number, %
Age, mean (SD)	61 [14]
Female sex	58
**Comorbidities**	
Hypertension	56
Diabetes mellitus	33
Coronary artery disease	22
Chronic obstructive pulmonary disease	9
Asthma	13
Chronic kidney disease (Cr > 2 mg/dL)	13
Malignancy	15
**Triage Vital Signs**	
sBP (SD), mmHg	141 [25]
dBP, mean (SD), mmHg	75 [14]
HR, mean (SD), beats/min	83 [19]
SpO_2_, mean (SD)	97.4 (3.5)
Temperature, mean (SD), °C	36.7 (0.5)
RR, *estimated*, mean (SD), breaths/min	18.4 (1.6)

Triage capnography measurements and outcomes are shown in Table 2. Among the 100 patients who had subsequent laboratory data, all had either a total bicarbonate or blood gas; however, 96 had only a total bicarbonate $\left[HC{O}_{3}^{-}\right]$ level and 53 had both a blood gas (*pH, P*_*v*_*CO*_*2*_) and bicarbonate for characterization of acid-base status. Tables 3 and 4 describe the breakdown of *RR* (slow, normal, fast) and *EtCO*_*2*_ (low, normal, high) and associated outcomes (acidemia, alkalemia, admission). Chi-square statistics calculated for both *RR* and *EtCO*_*2*_ were found to be statistically significant for acidemia and admission, but not for alkalemia.

**Table 2:** Triage capnography, laboratory values, and outcomes

	Included (*n* = 100)
Triage Capnography	
RR, *measured*, mean (SD), breaths/min	20.3 (6.4)
EtCO_2_, mean (SD), mmHg	36 (6.6)
**Laboratory Data**	
HCO_3_^−^ (SD), mg/dL	23.7 (3.8)
pH (SD)	7.38 (0.06)
**Outcomes**	
Acidemia (%)	28
Alkalemia (%)	8
Septic Shock (%)	2
Admission (%)	40
In-hospital Mortality (%)	2

*Abbreviations* systolic blood pressure (sBP), diastolic blood pressure (dBP), heart rate (HR), peripheral oxygen saturation (SpO_2_), respiratory rate (RR)

**Table 3:** RR stratification

		n	Acidemia	Alkalemia	Admission
**Resp Rate (bpm)**	**Slow (< 16)**	30	4	3	7
	**Normal**	34	11	2	14
	**Fast (> 22)**	36	13	3	19
	**Total**	100	28	8	40

*Abbreviations* respiratory rate (RR), end-tidal CO_2_ (EtCO_2_), total bicarbonate (HCO_3−_)

**Table 4:** EtCO_2_ stratification

		n	Acidemia	Alkalemia	Admission
**EtCO**_**2**_ **(mmHg)**	**Low (< 32)**	35	21	1	24
	**Normal**	35	5	2	12
	**High (> 40)**	30	2	5	4
	**Total**	100	28	8	40

**Fig. 2:**
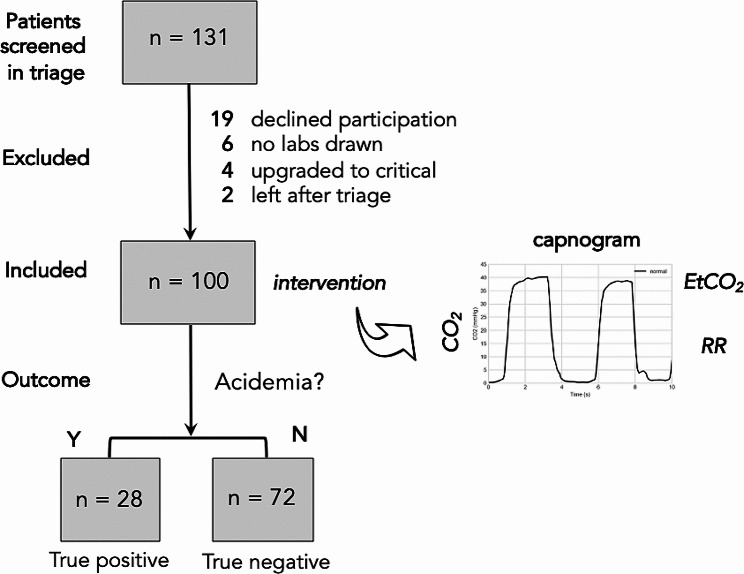
Flow diagram of study, including intervention comprising a 10-second capnogram, and laboratory determination of acidemia

**Fig. 3:**
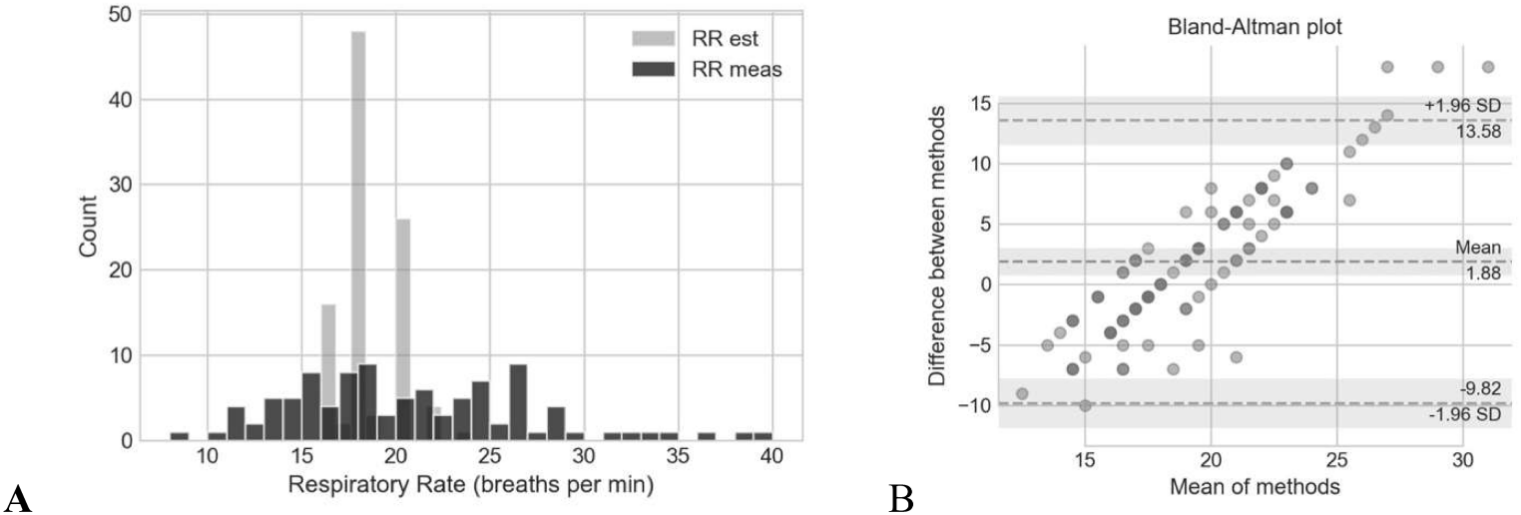
**A**. Histogram comparing frequency of measured versus estimated respiratory rates. Level of agreement between estimated and measured *RR* as summarized by (Fig. 3B) Bland-Altman analysis

The main findings of the study are summarized by the flow diagram in Fig. 2. Among the patients diagnosed with and without acidemia on laboratory measurement (true positives and true negatives, respectively), it is possible to construct a receiver-operator curve to determine optimal cutoffs for the test characteristics (summarized in Table 5). A histogram of measured and estimated *RR* is shown in Fig. 3A. Based on the Wilcoxon signed-rank test, the measured and estimated *RR* were found to be significantly different (*p = 0.006*).

**Table 5:** Diagnostic Accuracy for detecting acidemia

	Sensitivity (95% CI)	Specificity (95% CI)	LR+ (95% CI)	LR- (95% CI)	PPV (95% CI)	NPV (95% CI)
**RR > 25 bpm**	35.7 (18.6–55.9)	83.3 (72.7–91.1)	2.14 (1.05–4.39)	0.77 (0.57–1.04)	45.5 (28.9–63.1)	76.9 (71.3–81.7)
**EtCO**_**2**_ **< 32 mmHg**	71.43 (51.3–86.8)	84.7 (74.3–92.1)	4.68 (2.59–8.45)	0.34 (0.19–0.61)	64.5 (50.1–76.7)	88.4 (80.8–93.3)

**Fig. 4:**
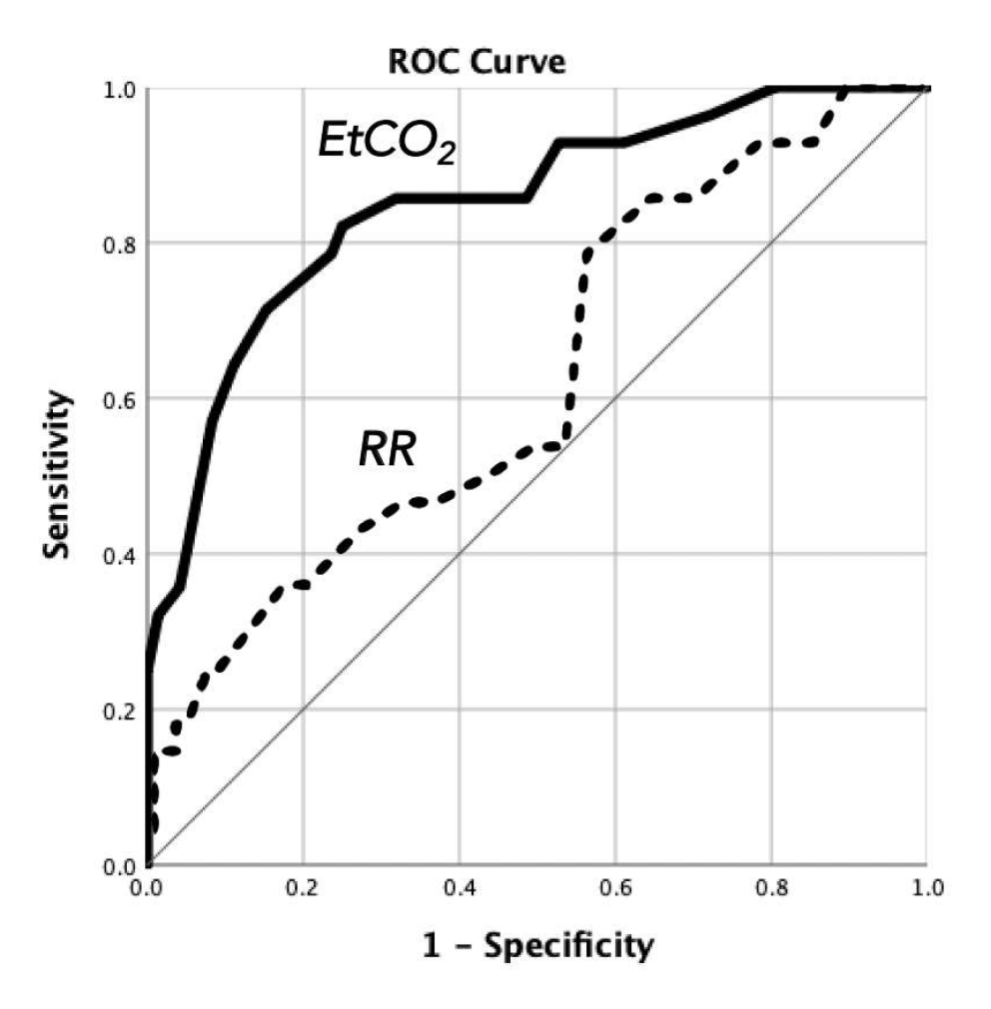
Receiver operating curves of *EtCO*_*2*_ (c = 0.85, 95% CI 0.76–0.94, *p* = 0.00) and *RR* (c = 0.6, 95% CI 0.51–0.75, *p* = 0.03) for predicting acidemia

The agreement between measured and estimated *RR* was analyzed using a Bland-Altman plot (Fig. 4) [21]. The measured *RR* was, on average, 1.88 breaths/min greater than that of estimated *RR*. The lack of agreement is quantified by the 95% CI of the differences, which was between − 9.8 and 13.6.

Accuracy of *RR* and *EtCO*_*2*_ for predicting acidemia was calculated to be 70.0% and 81.0%, respectively, based on a 28% prevalence. Splitting the data into a training and test datasets, we also modeled the combined predictive accuracy of *RR* and *EtCO*_*2*_ to be 83.3% using logistic regression.

**Fig. 5:**
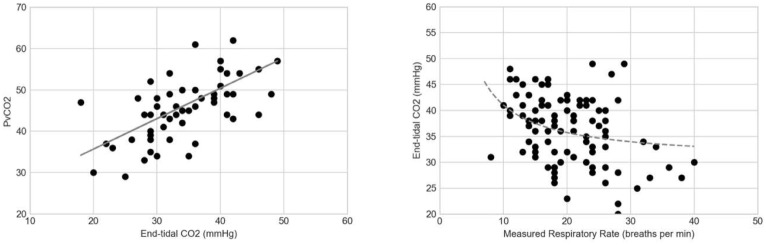
Relationship between *EtCO*_*2*_ and venous partial pressure of carbon dioxide (*P*_*v*_*CO*_*2*_), (R^2^ = 0.371) and *EtCO*_*2*_ vs. measured respiratory rate (R^2^ = 0.134)

Linear relationship between the venous partial pressure of carbon dioxide (*P*_*v*_*CO*_*2*_) and *EtCO*_*2*_ and inverse relationship between *EtCO*_*2*_ and measured respiratory rate are illustrated in Fig. 5. The accompanying best-fit lines were R^2^ = 0.371 for *P*_*v*_*CO*_*2*_ vs. *EtCO*_*2*_ and R^2^ = 0.134 for *EtCO*_*2*_ vs. measured respiratory rate.

Among the 28 patients diagnosed with acidemia, 22 were found with *low EtCO*_*2*_ (< 32 mmHg). The 22 ten-second capnogram waveforms are displayed in Supplemental Material, Fig. 2(A, B,C) along with brief chief complaint and inpatient diagnosis.

Approximately a quarter of the *low EtCO*_*2*_ cases have chief complaint of pain, illustrating the reason for decreased specificity of the capnogram for screening specific diseases. However, half of the chief complaints were notably secondary to renal or pulmonary processes, and the majority of capnograms with respiratory failure are associated with nearly absent alveolar plateau, largely from the rapid cycling of breaths. Of note, this study was completed one month before any presenting cases of COVID-19. No specific waveform feature, however, is characteristic of acidemia.

There were two cases of nonfatal opioid overdose—*after* administration of naloxone—with capnograms shown in Supplemental Material, Fig. 2A. Sometimes a capnogram can be the first indication of an unusual presentation: as shown in Supplemental Material, Fig. 1, a capnogram of a patient who had vital signs within normal limits (estimated *RR* = 20, measured *RR* = 26) was later discovered to have a both a moderate pericardial effusion and pleural effusion.

## Discussion

ED physicians are accustomed to capnography in the settings of achieving return of spontaneous circulation in cardiopulmonary resuscitation, confirming placement of endotracheal intubation, and monitoring respiration during procedural sedation [16]. Routine capnography in non-intubated patients beyond these applications has been considerably more limited [22–25]. A possible explanation to the limited use is that capnography is available only on certain monitors such that they would not be considered or used elsewhere. Another issue is cost, although portable capnometers can now be purchased for less than $2,000. There is also limited evidence regarding waveform interpretation, as there are no diagnostic criteria as to what constitutes an abnormal capnogram or *EtCO*_*2*_. Therefore, it is unsurprising that capnography has not been explored as a screening test. Yet this brief measurement could be a missed opportunity for an earlier, as well as noninvasive, detection of pathophysiologic processes if its test characteristics prove clinically useful.

Our pilot data among intermediate-risk adult patients presenting to the ED represent the initial steps to determine if an accurate determination of a triage capnogram—comprising *P*_*E*_*CO*_*2*_, *EtCO*_*2*_, and *RR—*can alert clinicians to acute processes or early decompensation. This study demonstrates that not only can *RR* be measured quickly and accurately, but also obtained along with *P*_*E*_*CO*_*2*_ and *EtCO*_*2*_. Additionally, these results corroborate other studies showing that a *low EtCO*_*2*_ may indicate an underlying acidosis.

Patients with underlying pulmonary disease have documented average respiratory rates up to + 11 breaths/min greater than the average, healthy adult [26]. A precisely-measured, elevated *RR* by itself could signify greater minute ventilation and respiratory drive, and this can be further corroborated by an *EtCO*_*2*_ level. Yet the prevailing standard of care is to rely on an estimated *RR*, which was shown to be in wide disagreement with the actual *RR*, over a range of -10 and 14 breaths/min. The present study suggests that capnograms could significantly improve triage in this patient population.

Our results indicate that a *low* end-tidal *CO*_*2*_ (*EtCO*_*2*_ < 32 mmHg) could represent an underlying acidosis—with positive likelihood ratios of 4.68 (95% CI 2.59–8.45). Assuming a pre-test odds of 0.39 based on a 28% prevalence of acidemia, ($o=\frac{p}{1-p}=\frac{0.28}{1-0.28}=0.39)$, the finding of *EtCO*_*2*_ < 32 mmHg has a post-test odds of ($o=\left(0.39\right)\left(4.68\right)=1.83$), which corresponds to a 65% post-test probability ($p=\frac{o}{1+o}\times 100\%=65\%$), roughly twice the pre-test probability. Unfortunately, the sensitivity 71.4% (95% CI 51.3-86.8) was also not sufficiently high enough to yield a negative likelihood ratio that is clinically useful to rule out acidemia. No study could be found in the literature among adult patients for comparison, but the utility of capnography has been more extensively investigated in pediatric patients. One study, for instance, demonstrated a correlation between *EtCO*_*2*_ < 31 mmHg and 96% specificity in detecting acidosis in cases of acute gastroenteritis [27]. This study of adult patients demonstrated that the specificity of acidosis was moderate, 84.7% (95% CI 74.3-92.1), indicating false positives that likely arose from increased respiratory drive secondary to pain [28]. Relatively poor correlation (R^2^ = 0.37) was found between *EtCO*_*2*_ and *P*_*v*_*CO*_*2*_ (Fig. 5). This has been observed in previous studies as well, and it is known that higher respiratory rates cause greater deviations [23].

The capnograms, while interpreted qualitatively, provide a rapid, visual perspective of the respiratory profile. The greater the steepness of the initial rise signifies better ventilation and perfusion (V/Q) matching; many pulmonary diseases have a slope with slower rise [29]. The next flat portion corresponds to the alveolar plateau, representing *P*_*E*_*CO*_*2*_ arising from alveoli. A wider angle, steeper plateau indicates slower CO_2_ elimination and differential alveolar ventilation. This was observed in the capnogram (Supplemental Material, Fig. 2A) of a patient who presented with an opioid overdose. While alveolar hypoventilation is expected to increase *EtCO*_*2*_, the measured capnograms are qualitatively similar to that of normal, healthy patients. This is not unexpected as respiratory drive was likely restored almost completely by naloxone. Poor V/Q matching also manifests as a greater angle between the rise and plateau.

The absence of an of alveolar plateau in the mixed expired gas *P*_*E*_*CO*_*2*_ (indicating rapid cycling) was found to be nearly always abnormal. The capnogram in Supplemental Material, Fig. 2A was highly unusual, including a rapid duty cycle, short alveolar plateau, *P*_*E*_*CO*_*2*_ not returning to *0* mmHg, and respirophasic variations. In this particular case, the patient was experiencing a severe asthma exacerbation, with short breaths indicative of obstructive ventilatory process. A short duty cycle is not specific, however, as patients experiencing severe pain (Supplemental Material, Fig. 2B) will also be tachypneic. Finally, waveforms with low amplitudes were also indicative of *EtCO*_*2*_ < 32 mmHg.

For true tachypnea (*RR > 25*), there is a positive likelihood ratio (LR+) of 2.14 (95% CI 1.05–4.39) of acidemia. If *RR* continues to be estimated, then it may be worthwhile to investigate the clinical impact of documenting rates as “slow”, “normal”, or “fast” instead, and to analyze the agreement with actual values. A continuation of this study would be to investigate the predictive value of triage *EtCO*_*2*_ on specific pathophysiologic processes, such as pneumonia or hypercapneic respiratory failure. It would also be interesting to study if merely a 10-second capnogram alone that was handed to a clinician like an electrocardiogram could lead to any significant changes in clinical management.

## Limitations

There are several limitations in this study. First, study subjects were derived from a convenience sample, introducing potential sampling bias towards a particular patient population; for example, 40% of study subjects were admitted to the hospital, which is significantly higher than the average admission rate (∼ 25%). Second, blood tests were drawn at variable times in relation to the time that capnography was performed. This time differential was not measured in this study, and longer delays would reduce the correlation to the acid-base status. Third, the limited duration of the study lead to a relatively small sample size (*n = 100*), which underpowers the detection of other disease processes—for instance, the prevalence of sepsis and opioid overdoses were surprisingly low (2%). Patient-centered outcomes such as in-hospital mortality were also not analyzed for the same reason. Finally, the respiratory profile was obtained only during triage, during which a patient could be in the most pain or anxious, and this could lead to falsely positive increased *RR*.

In conclusion, this pilot study demonstrates the feasibility of obtaining a rapid respiratory profile in triage, comprised of *P*_*E*_*CO*_*2*_, *EtCO*_*2*_, and *RR*. An accurate *RR* was significantly different and more clinically relevant than the traditional estimated value, and *EtCO*_*2*_ was more predictive of acidemia than *RR*. The utility of triage capnograms *P*_*E*_*CO*_*2*_ remains to be determined, but offers promise as a more accurate assessment of a patient’s ventilatory status at ED triage.

### Electronic supplementary material

Below is the link to the electronic supplementary material.

Supplementary Material 1

## Data Availability

All data gathered for this study are freely available upon request. The data are stored on encrypted drives and can be accessed by the primary author at the home institution.
