# Supplementary material for: Diagnostic utility of capnography in emergency department triage for screening acidemia: a pilot study
Source: Int J Emerg Med. 2024 Apr 22;17:57. doi: 10.1186/s12245-024-00631-3 (PMC11036727; doi:10.1186/s12245-024-00631-3)
Supplement: Supplementary file 1 — Supplementary Material 1 [file 12245_2024_631_MOESM1_ESM.docx]

Supplemental Materials, Figures

**Capnography Physiology**

Capnography, which yields the mixed expired gas, $P_{E}{CO}_{2}$, as a function of time (i.e. capnogram) along with carbon dioxide (${CO}_{2})$ at end-expiration, the end-tidal ${CO}_{2}$ (*EtCO_2_*). The expired gas closely approximates the alveolar partial pressure of carbon dioxide, $P_{A}{CO}_{2}$, which is in turn directly related to the metabolic production of ${CO}_{2}$ in the body, $\dot{V}{CO}_{2}$, and inversely related to the alveolar minute ventilation $\dot{V}_{A}$,

$$P_{A}{CO}_{2} \propto\frac{\dot{V}{CO}_{2}}{\dot{V}_{A}}$$

where $\dot{V}_{A}=RR\times V_{A}$ and $V_{T}=V_{A}+V_{D}$. Generally speaking, the expired gas, $P_{E}{CO}_{2}$, can be traced back to the carbon dioxide carried as bicarbonate or carbamino compounds in the blood, such that $P_{E}{CO}_{2}$ is at least a *lower* bound to the arterial tension of carbon dioxide. The arterial to end-tidal *CO_2_* gradient is approximately 2-5 mmHg and would increase with greater dead space.^2^ If physiologic dead space is negligible ($V_{D} \sim0$), then $V_{T}\approx V_{A}$ and $\dot{V}_{A}\sim RR\times V_{T}$ (minute ventilation):

$$P_{E}{CO}_{2} \propto\frac{\dot{V}{CO}_{2}}{RR\times V_{T}}$$

The above relationship is central to this study’s hypothesis that a capnograph respiratory profile (*RR*, $P_{E}{CO}_{2}$, *EtCO_2_*) could reflect an underlying derangement in physiology.

**
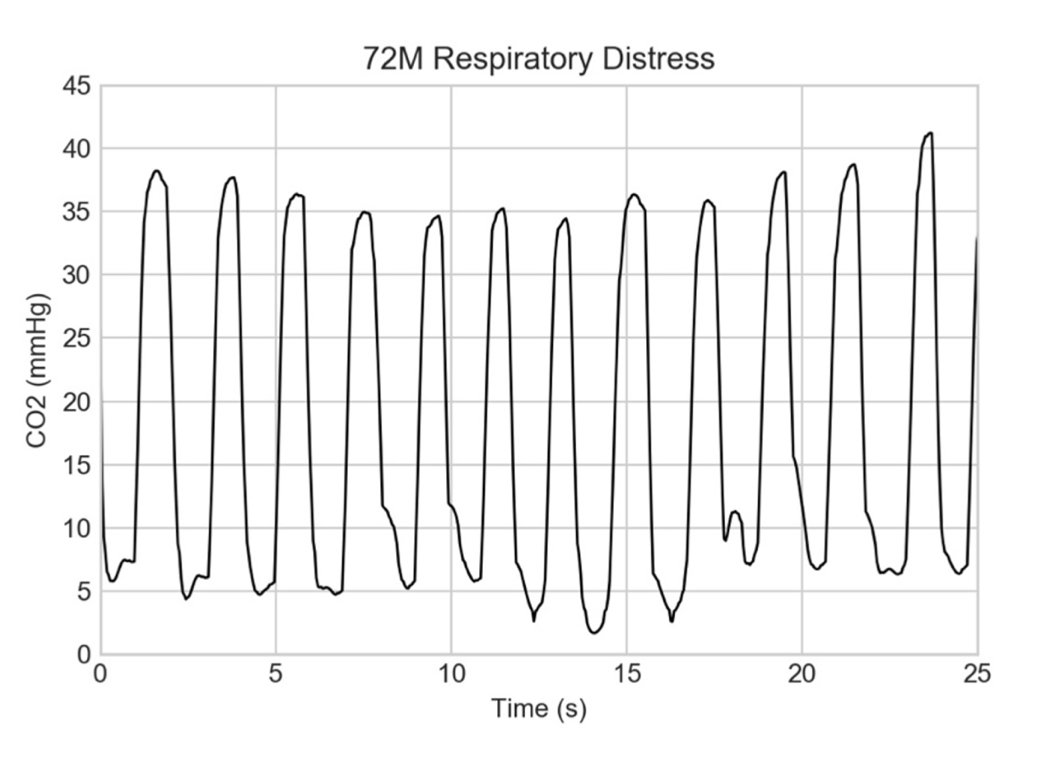
**

**Supplemental Figure 1** Unusual capnogram of patient later found with a large pericardial effusion

**(A)**


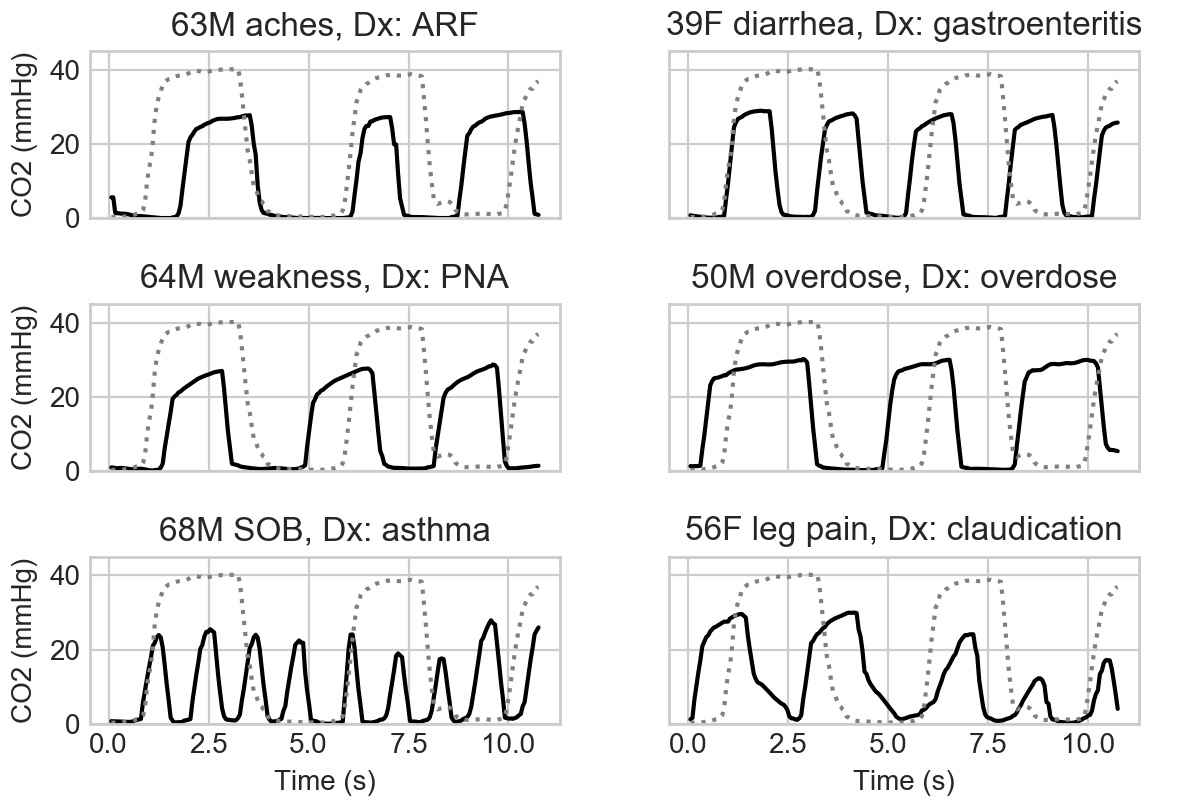


**(B)**


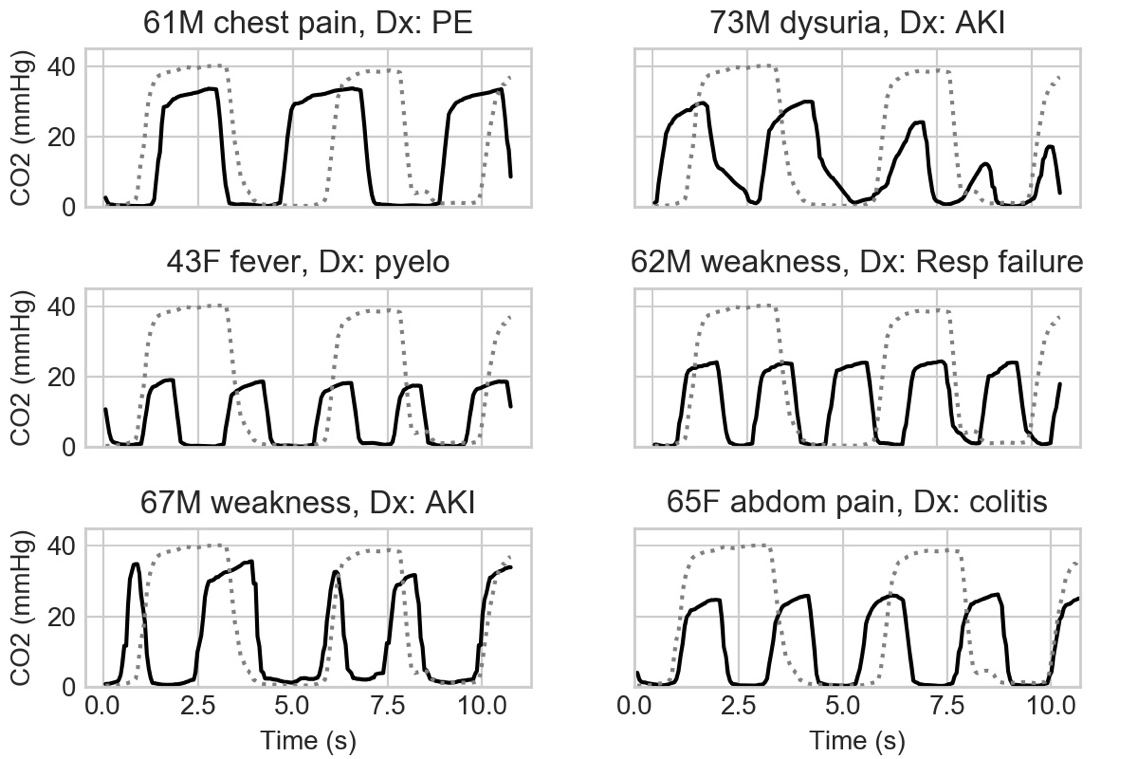


**(C)**


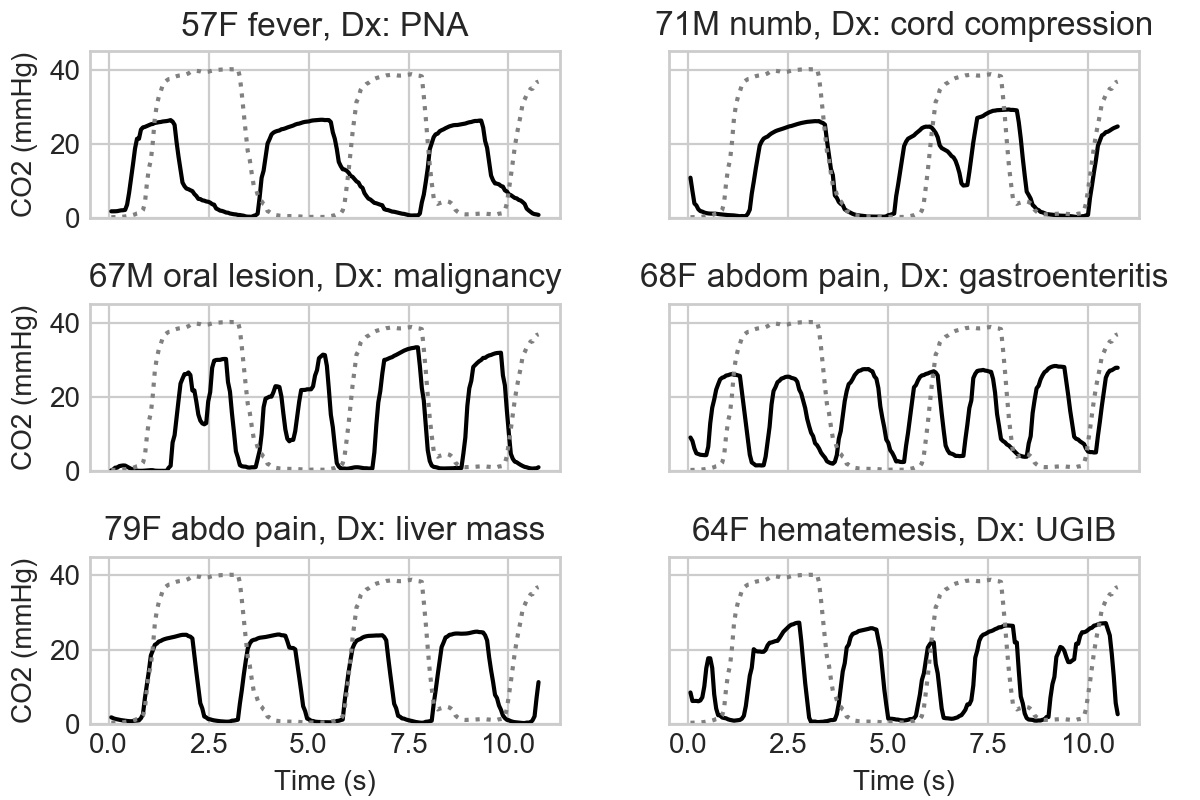


**
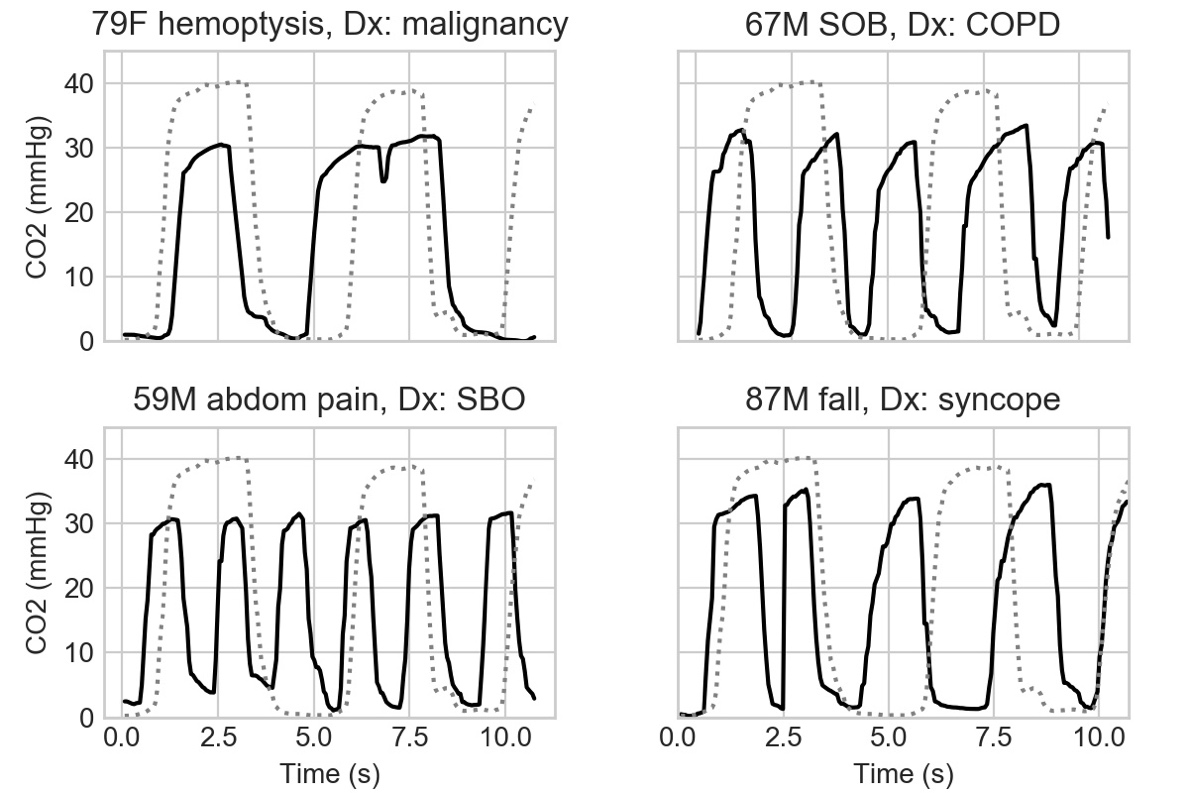
**

**Supplemental Figure 2** Capnograms of 22 patients
